# The health belief model and number of peers with internet addiction as inter-related factors of Internet addiction among secondary school students in Hong Kong

**DOI:** 10.1186/s12889-016-2947-7

**Published:** 2016-03-16

**Authors:** Yanhong Wang, Anise M. S. Wu, Joseph T. F. Lau

**Affiliations:** Department of Epidemiology and Bio-statistics, Institute of Basic Medical Sciences, Peking Union Medical College/China Academy of Medical Sciences, Beijing, China; Department of Psychology, Faculty of Social Sciences, University of Macau, Macao, China; Centre for Health Behaviours Research, JC School of Public Health and Primary Care, Faculty of Medicine, The Chinese University of Hong Kong, Hong Kong, China; The Chinese University of Hong Kong Shenzhen Research Institute, Shenzhen, China

**Keywords:** Internet addiction, Health belief model, Secondary school students

## Abstract

**Background:**

Students are vulnerable to Internet addiction (IA). Influences of cognitions based on the Health Belief Model (HBM) and perceived number of peers with IA (PNPIA) affecting students’ IA, and mediating effects involved, have not been investigated.

**Methods:**

This cross-sectional study surveyed 9518 Hong Kong Chinese secondary school students in the school setting.

**Results:**

In this self-reported study, the majority (82.6 %) reported that they had peers with IA. Based on the Chinese Internet Addiction Scale (cut-off =63/64), the prevalence of IA was 16.0 % (males: 17.6 %; females: 14.0 %). Among the non-IA cases, 7.6 % (males: 8.7 %; females: 6.3 %) perceived a chance of developing IA in the next 12 months. Concurring with the HBM, adjusted logistic analysis showed that the Perceived Social Benefits of Internet Use Scale (males: Adjusted odds ratio (ORa) = 1.19; females: ORa = 1.23), Perceived Barriers for Reducing Internet Use Scale (males: ORa = 1.26; females: ORa = 1.36), and Perceived Self-efficacy for Reducing Internet Use Scale (males: ORa = 0.66; females: ORa = 0.56) were significantly associated with IA. Similarly, PNPIA was significantly associated with IA (‘quite a number’: males: ORa = 2.85; females: ORa = 4.35; ‘a large number’: males: ORa = 3.90; females: ORa = 9.09). Controlling for these three constructs, PNPIA remained significant but the strength of association diminished (‘quite a number’: males: multivariate odds ratio (ORm) = 2.07; females: ORm = 2.44; ‘a large number’: males: ORm = 2.39; females: ORm = 3.56). Hence, the association between PNPIA and IA was partially mediated (explained) by the three HBM constructs. Interventions preventing IA should change these constructs.

**Conclusions:**

In sum, prevalence of IA was relatively high and was associated with some HBM constructs and PNPIA, and PNPIA also partially mediated associations between HBM constructs and IA. Huge challenges are expected, as social relationships and an imbalance of cost-benefit for reducing Internet use are involved. Perceived susceptibility and perceived severity of IA were relatively low and the direction of their associations with IA did not concur with the HBM. Group cognitive-behavioral interventions involving peers with IA or peers recovered from IA are potentially useful to modify the HBM constructs and should be tested for efficacy.

## Background

Internet addiction (IA) is defined as an impulse-control disorder of Internet use that has negative impacts on daily life function, family relationships, and emotional stability [1–3]. Contemporary adolescents grow up in an environment that Internet use has become increasingly important in multi-facets of life [4]. Adolescents tend to have lower self-control for online activities and are more likely than adults to develop IA [5]. The prevalence of IA among adolescents varies across countries [2], ranging from 1.9 to 8.2 % in European countries [6–9], from 2.3 to 20.7 % in Asian countries [10–15], and from 6.7 to 26.7 % in Hong Kong [16–18]. The variation is possibly due to different methodologies (e.g., measurement tools, mode of data collection, sampling methods) and time-frames used in these studies [18]. Among adolescents, IA has been associated with psychosocial problems, including poor academic performance, poor parental-child relationships, physical and mental health problems, withdrawal from daily life, hostility, and aggressiveness [2, 5, 17, 19–21]. Previous studies conducted in Asia (e.g., Taiwan [20–25], Hong Kong [16–18], South Korea [11, 26, 27], and mainland China [13, 28]) reported significant risk factors of IA, including sexual intercourse experience, family problems, low self-esteem, social isolation, impulsivity, Internet accessibility and other risk behaviors [12, 27, 29–32].

As theory-based interventions are more effective than non-theory-based ones [33], it is warranted to apply such theories to understand IA. The Health Belief Model (HBM), a commonly used theory, has been applied to explain various health-related behaviors (e.g., condom use [34], tooth brushing [35], and motorcycle helmet use [36]) and to design related interventions (e.g., influenza vaccination [37] and helmet promotion program [38]) among adolescents. It consists of six constructs: perceived susceptibility, perceived severity, perceived benefits, perceived barriers, cue to action and self-efficacy [39, 40]. According to the model, adolescents who are not threatened by IA (those who perceive low susceptibility and low severity), see good reasons to use the Internet (high perceived benefits), expect adverse consequences from reducing Internet use (high perceived barriers), find it difficult to reduce Internet use (low perceived self-efficacy to reduce Internet use), and are seldom reminded by others to reduce Internet use (absence of cue to action), are more likely than others to develop IA. This important theory has however, not been applied to understand IA among both adolescents and adults.

Furthermore, peer influence is one of the strongest determinants of risk behaviors among adolescents [41–45], as they tend to respond strongly to social reward and conformity [46]. According to the Theory of Planned Behavior, supportive subjective norm, which is defined as significant others’ approval of a particular risk behavior, is an important determinant of the risk behavior [47]. In addition, affiliation with peers engaging in a particular risk behavior is a risk factor of that behavior (e.g., substance abuse, smoking, and alcohol drinking [41–45, 48]). Such an association can be explained by processes such as social pressure [47], social selection [41, 49, 50] and social learning [51, 52]. Previous studies have reported that affiliations with peers who use alcohol, illicit drugs, or have other deviant behaviors were risk factors of IA among adolescents [19, 53], but the impact of the presence of peers with IA remains unknown. We contend that affiliation with peers who have IA is a potential risk factor of IA among adolescents. While the aforementioned types of risk behaviors are regarded by the public as harmful, undesirable misconduct, or even illegal, Internet use by itself could be neutral or even seen as beneficial in the eyes of some adolescents. Hence, peer influences on IA, which have not been well-studied, may be different from those of other risk behaviors. Research is warranted to clarify the relationship between peer influence and IA.

It is important to investigate the underlying mechanism behind the association between affiliation with peers with IA and IA among adolescents. According to the Peer Cluster Theory [54], peers are strong socializing agents who actively shape other adolescents’ behaviors by changing their corresponding beliefs and attitudes. Peers with behavioral problems may serve as negative role models [55], hence adolescents may directly learn and adopt favorable beliefs of Internet use and IA (e.g., low perceived severity of IA and high perceived benefits of Internet use) from peers with IA [56]. Moreover, observing peers with IA who have failed to control their Internet use may lower one’s perceived self-efficacy on controlling Internet use through vicarious learning [57], thus heightening his/her risk of developing IA. Therefore, cognitions related to the HBM are potential mediators of the association between affiliation with peers with IA and IA among adolescents.

In the present study, we investigated the prevalence of IA among Chinese secondary school students in Hong Kong, China. Two categories of associated factors were investigated: 1) cognitive factors based on the HBM, and 2) perceived number of peers with IA (PNPIA). We hypothesized that the association between PNPIA and IA, if any, would be mediated by some HBM factors. This is the first study investigating the role of the HBM to understand IA, including its direct and mediating effects.

## Methods

### Study design

A cross-sectional survey was conducted from September 2012 to January 2013. One school was randomly selected from each of the 19 districts in Hong Kong and participants were evenly recruited from all Chinese Secondary 1 to 4 (i.e., 7–10th year of formal education) students of such schools. Secondary 5 and 6 students were not included because they needed to prepare for university entrance examinations and thus some selected schools did not recommend those students to join the survey. It is a limitation that age of the participants was not asked in this study because we expected the participants to have a relatively narrow age range. Age was strongly associated with school grade and in Hong Kong, Secondary 1 to 4 students range from 12 to 16 years old [58]. The participants were briefed by fieldworkers and filled out an anonymous and structured questionnaire in classroom settings, in the absence of teachers. They were guaranteed that the collected data would only be accessed by the researchers. Written consent was obtained from parents (average response rate of the school = 94.1 %; SD = 4.1 %). Of the 9,666 students who filled out the survey, 148 (1.53 %) were excluded from data analysis due to incomplete questionnaires. No incentive was provided to the participants. Ethics approval was obtained from the Ethics Committee of the Chinese University of Hong Kong.

### Measures

Information on socio-demographics (including grades, parents’ education attainment, father’s age, living arrangement with parents, place of birth, and duration of residency in Hong Kong) was collected. PNPIA was assessed by the question: “How many friends of yours do you think have IA?” Responses included *nil, only a few, quite a number,* and *a large number*.

The 26-item Chinese Internet Addiction Scale (CIAS) was used to identify probable cases of IA [59]. It has five constructs, including symptoms of compulsive use, withdrawal, tolerance, problems in interpersonal relationships and physical conditions, and time management [59]. The item responses range from “*definitely disagree*” (1) to “*definitely agree*” (4). A summative score was formed, with higher scores indicating higher severity of IA (range =26 to 104). The original instrument showed good psychometric properties [59], and has been used among Chinese adolescents in Hong Kong [16] and Taiwan [21, 25, 60–62]. Using the cut-off value of 63/64 [63], CIAS yielded high specificity of 92.6 % and an excellent diagnostic accuracy rate of 87.6 % in another study [63]. In this study, the Cronbach’s alpha of the total scale was 0.95. Cronbach’s alpha is the most widely used method for assessing reliability of measurement tools [64]. It has been reported in many studies, including those on IA [18, 20]. Its highest value is 1.0, and values 0.5 to 0.6 are regarded as acceptable in preliminary research [65].

We constructed six scales for the six constructs of the HBM on issues related to Internet use and IA, with a 5-point Likert response scale (from *definitely disagree* [1] to *definitely agree* [5]). A panel was formed, including psychologists, public health researchers, and epidemiologists, to create items of such scales. (1) *Perceived Susceptibility to IA* was assessed by a single item: “You will have IA in the next 12 months”. (2) *Perceived Severity of IA* was assessed by the item: “The consequences would be very harmful if I had IA”. (3) The *Perceived Social Benefits of Internet Use Scale* summed up the item scores of five items assessing perceived social benefits (“The Internet is an important means to keep in touch with my friends in real life”, “The Internet is the key for me to meet new friends”, “On the Internet, there is someone I trust who can solve my problems”, “On the Internet, there is someone who can give me advice when I need to make important decisions”, and “On the Internet, there is someone who can talk to me when I feel lonely”). Cronbach’s alpha was 0.80 (range = 5 to 25). (4) *The Perceived Barriers for Reducing Internet Use Scale* involved four items (Reduction of Internet use would result in: ‘less communication with your friends’, ‘feeling lost’, ‘a boring life’, or ‘feeling old fashion’). The summative scale ranged from 4 to 20, with a Cronbach’s alpha of 0.82. (5) The *Cue to Action to Reduce Internet Use Scale* involved two statements (“Your parents have often asked you to reduce Internet use” and “Your teachers/social workers have asked you to reduce Internet use”). The summative scale scores ranged from 2 to 10 (Cronbach’s alpha = 0.58). (6) Lastly, the *Perceived Self-efficacy for Reducing Internet Use* involved two items: “You are confident to reduce Internet use if you would like to do so”, and “You find it difficult to reduce Internet use”. The summative scale was formed by summing up the score of the first item and the reversed score of the second item; it ranged from 2 to 10 (Cronbach’s alpha = 0.63). Higher scores of the constructed scales represented higher levels of the constructs.

### Statistical analysis

The dependent variable was probable IA, defined as having a CIAS score >63 [63]. Chi-square test and *t*-test were used to compare between-group gender differences on all independent variables (including socio-demographic factors, HBM constructs, and PNPIA), and separate analyses were conducted for males and females. Spearman correlation coefficients between PNPIA and the significant scales that concurred with the HBM were presented. Univariate odds ratios (ORu) were firstly derived for all independent variables. Adjusted odds ratios (ORa) were then obtained for the HBM constructs and PNPIA by fitting multiple logistic regression models, adjusted for all socio-demographic variables that were found to be statistically significant (*p* < 0.05) in the univariate analysis. Lastly, a multiple logistic regression model was fit to derive multivariate odds ratios (ORm) by entering PNPIA and those scales that were significantly associated with IA and concurred with the HBM into the same model. The model was also adjusted for socio-demographic variables that were found to be statistically significant in the univariate analysis. Respective 95 % confidence intervals (CI) were derived for all odds ratios presented in the report. Statistical significance was defined as *p* < 0.05 and all analyses were conducted by using SAS 9.2 (SAS Institute, Cary, NC, USA).

## Results

### Background characteristics of the participants

Among all students, the majority lived with both parents (82.7 %) and were born in Hong Kong (78.6 %), and 16.6 and 13.3 % of the participants’ father and mother had attended universities, respectively. The male and female distributions of socio-demographic factors were comparable (Table 1). Although some statistically significant differences were detected due to the very large sample sizes, such differences were usually smaller than 2 to 3 % and did not seem to carry practical significance.

**Table 1:** Participants’ characteristics by sex^a^

	Males	Females	Total	*p* value*
	(*N* = 5062)	(*N* = 4396)	(*N* = 9518)	
	%/mean (SD)	%/mean (SD)	%/mean (SD)	
School grade				
Secondary 1	24.71	23.23	23.98	0.043
Secondary 2	24.71	24.25	24.51	
Secondary 3	26.49	26.00	26.31	
Secondary 4	24.08	26.52	25.20	
Father’s education level				
Junior secondary school or below	26.51	29.50	27.83	<0.001
Senior secondary school or matriculation	37.34	39.04	38.22	
University or college or above	18.25	14.70	16.57	
Missing	17.90	16.77	17.38	
Mother’s education level				
Junior secondary school or below	27.12	30.48	28.66	<0.001
Senior secondary school or matriculation	40.34	43.06	41.67	
University or college or above	15.27	11.03	13.25	
Missing	17.27	15.42	16.42	
Living arrangement with parents				
Living with both parents	82.83	82.50	82.70	0.143
Living with the mother only	9.25	9.92	9.55	
Living with the father only	3.14	2.82	2.99	
Living with none of the parents	2.67	3.34	2.97	
Missing	2.11	1.39	1.79	
Father’s age (mean ± SD)	46.95 ± 6.78	47.08 ± 6.59	47.02 ± 6.68	0.428
Whether born in HK/length of residency				
Yes, born in HK	79.89	77.05	78.58	<0.001
No, stayed in HK ≥7 years	11.81	13.33	12.53	
No, stayed in HK <7 years	5.55	7.48	6.43	
No, can’t remember when came to HK	2.00	1.66	1.84	
Missing	0.75	0.48	0.62	
HBM constructs				
Perceived Susceptibility to IA	2.36 ± 1.08	2.35 ± 1.00	2.36 ± 1.04	0.558
Perceived Severity of IA	3.29 ± 1.26	3.52 ± 1.12	3.40 ± 1.20	<0.001
Perceived Barriers (reducing use)	10.39 ± 3.99	10.81 ± 3.82	10.59 ± 3.92	<0.001
Perceived Social Benefits (Internet use)	13.70 ± 4.20	13.40 ± 3.99	13.57 ± 4.11	<0.001
Cue to Action Scale (reducing use)	5.46 ± 2.05	4.91 ± 1.95	5.21 ± 2.03	<0.001
Perceived Self-efficacy (reducing use)	7.08 ± 2.04	7.30 ± 1.90	7.18 ± 1.98	<0.001
Perceived number of peers with IA (PNPIA)				
Nil	16.69	18.08	17.35	<0.001
Only a few	41.21	52.30	46.34	
Quite a number	29.26	24.57	27.10	
A large number	12.84	5.05	9.21	

Cases with missing information for gender were excluded from analysis (*n* = 60)

*χ^2^ test or 2-sample t-test

^a^Figures for the variable on school attended by participants (18 schools) were not listed in this table

### Distribution of PNPIA

The percentage distribution of the variable on PNPIA was: 17.4 % for *nil* (male: 16.7 %, female: 18.1 %), 46.3 % for *only a few* (male: 41.2 %, female: 52.3 %), 27.1 % for *quite a number* (male: 29.3 %, female: 24.6 %), and 9.2 % for *a large number* (male: 12.8 %, female: 5.1 %). Male students reported significantly higher PNPIA than female students (*p* < 0.001; Table 1).

### Prevalence of IA

About one sixth of the participants (16.0 %; 95 % CI: 15.2 to 16.7 %) were classified as cases of IA (i.e., CIAS score > 63), and the IA prevalence of male students (17.6 %; 95 % CI: 16.6 to 18.7 %) was slightly higher than that of female students (14.0 %; 95 % CI: 13.0 to 15.1 %; *p* < .001). IA prevalence broken down by both participants’ school grade and sex is presented in Table 2. The mean CIAS score was 50.2 (SD = 14.6) for males and 49.2 (SD = 13.9) for females. The CIAS scores were not normally distributed according to Kolmogorov-Smirnov test both for males (*p* < 0.01) and females (*p* < 0.01), but our logistic regression analysis was not affected because our dependent variable of IA was binary and such analysis does not require normal distribution for the dependent variable.

**Table 2:** Associations between socio-demographic factors and IA^a,b^

	Males		Females	
	%	OR_u_ (95 % CI)	%	OR_u_ (95 % CI)
School grade				
Secondary 1	12.23	1.00	9.30	1.00
Secondary 2	17.83	1.56 (1.25 ~ 1.95)	13.88	1.57 (1.20 ~ 2.07)
Secondary 3	19.69	1.76 (1.42 ~ 2.18)*	14.61	1.67 (1.28 ~ 2.18)
Secondary 4	20.75	1.88 (1.51 ~ 2.34)**	17.67	2.09 (1.61 ~ 2.71)**
Father’s education level				
Junior secondary school or below	21.76	1.00	15.34	1.00
Senior secondary school or matriculation	17.14	0.74 (0.62 ~ 0.89)**	13.81	0.88 (0.72 ~ 1.08)
University or college or above	12.34	0.51 (0.40 ~ 0.64)**	12.69	0.80 (0.61 ~ 1.06)
Missing	17.99	0.79 (0.64 ~ 0.98)*	13.30	0.85 (0.65 ~ 1.10)
Mother’s education level				
Junior secondary school or below	23.09	1.00	15.52	1.00
Senior secondary school or matriculation	16.01	0.64 (0.54 ~ 0.76)**	13.63	0.86 (0.71 ~ 1.05)
University or college or above	12.68	0.48 (0.38 ~ 0.62)**	10.72	0.65 (0.47 ~ 0.90)*
Missing	17.28	0.70 (0.56 ~ 0.86)**	14.45	0.92 (0.71 ~ 1.19)
Living arrangement with parents				
Living with both parents	16.46	1.00	13.09	1.00
Living with the mother only	22.44	1.47 (1.16 ~ 1.85)**	18.58	1.52 (1.17 ~ 1.96)**
Living with the father only	30.82	2.26 (1.60 ~ 3.20)**	20.97	1.76 (1.13 ~ 2.74)*
Living with none of the parents	20.00	1.27 (0.83 ~ 1.95)	17.01	1.36 (0.88 ~ 2.11)
Missing	20.56	1.31 (0.82 ~ 2.12)	14.75	1.15 (0.56 ~ 2.35)
Father’s age	Not applicable	1.01 (0.99 ~ 1.02)	Not applicable	1.01 (0.99 ~ 1.02)
Whether born in HK/length of stay				
Yes, born in HK	17.01	1.00	13.88	1.00
No, stayed in HK ≥7 years	20.74	1.28 (1.03 ~ 1.58)*	15.02	1.10 (0.86 ~ 1.40)
No, stayed in HK <7 years	19.93	1.21 (0.90 ~ 1.65)	13.37	0.96 (0.69 ~ 1.34)
No, don’t remember when came to HK	16.83	0.99 (0.58 ~ 1.67)	12.33	0.87 (0.43 ~ 1.77)
Missing	21.05	1.30 (0.59 ~ 2.85)	23.81	1.94 (0.71 ~ 5.32)

*p value < 0.05; **p value < 0.01

^a^CIAS > 63

^b^Figures for the school variable were not listed in this table

OR_u_: univariate odds ratios

### Perceived susceptibility and perceived severity related to IA

Slightly more than one tenth (12.2 %) of all the sampled students (13.3 % for males and 10.9 % for females, *p* < 0.001) either definitely agreed or agreed that they would develop IA in the next 12 months (perceived susceptibility). Regarding perceived severity, only 54.7 % of all participants either definitely agreed or agreed that IA would be harmful to them (50.7 % for males and 59.2 % for females; *p* < 0.001; Table 3). Among the IA cases, the prevalence of perceived susceptibility was 36.5 % (35.2 % for males and 38.8 % for females, *p* < 0.001), and 7.6 % among non-IA cases (8.7 % for males; 6.3 % for females, *p* < 0.001; data were not tabulated). Only about half of the IA cases (50.2 % for males and 56.8 % for females, *p* = 0.039) and about 55.0 % of the non-IA cases (50.9 % for males and 59.6 % for females, *p* < 0.001) perceived that IA would be harmful to them (data not tabulated). Comparisons of perceived IA susceptibility and severity between IA and non-IA cases are made in a later section of this report.

**Table 3:** Frequencies of items based on constructs of the HBM by sex

	Males			Females			Total		
	Disagree (%)	Neutral (%)	Agree (%)	Disagree (%)	Neutral (%)	Agree (%)	Disagree (%)	Neutral (%)	Agree (%)
Perceived Susceptibility to IA	52.71	33.96	13.33	53.82	35.33	10.85	53.21	34.59	12.20
Perceived Severity of IA	26.02	23.25	50.73	18.81	21.95	59.24	22.66	22.65	54.69
Perceived Barriers (reducing Internet use)									
Reduced communication with your friends	52.09	18.87	29.04	50.43	15.83	33.74	51.31	17.44	31.25
Feeling lost	54.13	22.17	23.71	55.32	21.41	23.27	54.65	21.80	23.54
Feeling bored	48.26	19.60	32.14	47.00	16.90	36.10	47.64	18.33	34.03
Feeling old fashion	54.13	21.08	24.79	47.59	20.38	32.03	51.02	20.73	28.25
At least one of above = “agree”	N.A.	N.A.	50.97	N.A.	N.A.	57.48	N.A.	N.A.	54.07
Perceived Social Benefits (Internet use)									
To keep in touch with friends	28.17	39.41	32.42	29.05	36.03	34.92	28.52	37.84	33.63
To meet new friends	60.71	27.46	11.83	67.70	21.00	11.31	63.97	24.46	11.57
Someone who helps solving problems	37.36	39.00	23.65	43.70	38.49	17.81	40.24	38.80	20.96
Someone who gives advices	33.98	34.18	31.85	37.19	35.17	27.64	35.46	34.61	29.93
Someone to talk to	32.38	35.72	31.90	33.51	32.94	33.55	32.85	34.50	32.64
At least one of above = “agree”	N.A.	N.A.	58.42	N.A.	N.A.	58.78	N.A.	N.A.	58.65
Cue to Action (reducing Internet use)									
Reminded by parents	34.33	18.47	47.19	45.50	16.26	38.24	39.45	17.43	43.12
Reminded by teachers/social workers	54.11	32.93	12.96	65.58	26.68	7.73	59.35	30.06	10.59
Perceived Self-efficacy (reducing Internet use)									
Confidence to reduce Internet use	17.72	29.95	52.33	11.99	29.41	58.60	15.06	29.71	55.23
Difficulty to reduce Internet use	53.54	27.40	19.06	58.55	25.36	16.08	55.87	26.40	17.72

NA: Not applicable

### Other HBM cognitions related to Internet use

Over half of the agreed with at least one item in the Perceived Barriers for Reducing Internet Use Scale (54.1 % for all students; 51.0 % for males; 57.5 % for females) or Perceived Social Benefits of Internet Use Scale (58.7 % for all students; 58.4 % for males; 58.8 % for females). Among the IA cases, the percentages of agreeing with at least one item was 79.3 % (74.5 % for males and 86.2 % for females) in the Perceived Barriers for Reducing Internet Use Scale, and 77.4 % (74.6 % for males and 81.2 % for females) in Perceived Social Benefits of Internet Use Scale (data not tabulated). Regarding cue to action, 43.1 and 10.6 % of the participants reported that their parents and teachers/social workers had suggested them to reduce Internet use, respectively. Among the IA cases, however, nearly one-third had neither been reminded by their parents, nor by their teachers/social workers (32.7 % for all, 31.0 % for males, and 35.4 % for females; data not tabulated). Regarding perceived self-efficacy to reduce Internet use, 15.1 % (17.7 % for males; 12.0 % for females) agreed that they were not confident to reduce their Internet use, and 17.7 % agreed that they perceived difficulty in reducing their Internet use (19.1 % for males and 16.1 % for females, Table 3). Females, as compared to males, scored significantly higher in perceived severity, perceived barriers, and perceived self-efficacy, but scored lower in perceived social benefits and cue to action (Table 1).

### Socio-demographic factors associated with IA stratified by sex

In the univariate analysis (Table 2), socio-demographic factors that were significantly associated with IA among male students included: 1) higher form (Secondary 2: 17.8 % and ORu = 1.56; Secondary 3: 19.7 % and ORu = 1.76; Secondary 4, 20.8 % and ORu = 1.88; reference group = Secondary 1: 12.2 %) and 2) not living with both parents (living with mother only: ORu = 1.47; living the father only: ORu = 2.26), while higher paternal education level (senior secondary school or matriculation, ORu = 0.74; university or college or over, ORu = 0.51) and higher maternal education level (senior secondary school or matriculation, ORu = 0.64; university or college or over, ORu = 0.48) were protective factors against IA. Similar significant factors were found for females, except for paternal education level which was found to be non-significant. The odds ratios for females are summarized in Table 2.

### HBM constructs and PNPIA as factors of IA

Adjusted for the aforementioned significant socio-demographic factors, with one exception (perceived severity for female students), the six variables (scales) based on the HBM were all significantly associated with IA for both male and female students (Table 4).

**Table 4:** Factors (three specific HBM constructs and PNPIA) associated with IA

	Male students			Female students		
	OR_u_ (95 % CI)	OR_a_ (95 % CI)	OR_m_ (95 % CI)	OR_u_ (95 % CI)	OR_a_ (95 % CI)	OR_m_ (95 % CI)
Constructs of the HBM						
Perceived Susceptibility to IA	2.31 (2.14 ~ 2.50)**	2.27 (2.10 ~ 2.46)**	N.A.	3.40 (3.05 ~ 3.79)**	3.41 (3.05 ~ 3.82)**	N.A.
Perceived Severity of IA	1.07 (1.01 ~ 1.13)*	1.14 (1.07 ~ 1.21)**	N.A.	0.98 (0.91 ~ 1.06)	1.01 (0.94 ~ 1.10)	N.A.
Perceived Barriers (reducing Internet use)	1.26 (1.23 ~ 1.28)**	1.26 (1.23 ~ 1.29)**	1.15 (1.12 ~ 1.18)**	1.36 (1.32 ~ 1.40)**	1.36 (1.32 ~ 1.40)**	1.20 (1.16 ~ 1.24)**
Perceived Social Benefits (Internet use)	1.20 (1.17 ~ 1.22)**	1.19 (1.17 ~ 1.22)**	1.10 (1.07 ~ 1.12)**	1.23 (1.20 ~ 1.26)**	1.23 (1.20 ~ 1.26)**	1.10 (1.07 ~ 1.13)**
Cue to Action (reducing Internet use)	1.36 (1.31 ~ 1.42)**	1.36 (1.30 ~ 1.41)**	N.A.	1.43 (1.36 ~ 1.50)**	1.47 (1.40 ~ 1.55)**	N.A.
Perceived Self-efficacy (reducing Internet use)	0.66 (0.64 ~ 0.69)**	0.66 (0.64 ~ 0.69)**	0.76 (0.73 ~ 0.80)**	0.56 (0.53 ~ 0.59)**	0.56 (0.53 ~ 0.59)**	0.67 (0.63 ~ 0.72)**
Perceived number of peers with IA (PNPIA)						
Nil	1.00	1.00	1.00	1.00	1.00	1.00
Only a few	0.95 (0.73 ~ 1.22)	1.02 (0.78 ~ 1.33)	0.91 (0.69 ~ 1.21)	1.53 (1.12 ~ 2.09)**	1.50 (1.10 ~ 2.06)**	1.30 (0.92 ~ 1.82)
Quite a number	2.78 (2.17 ~ 3.55)**	2.85 (2.21 ~ 3.67)**	2.07 (1.57 ~ 2.73)**	4.44 (3.24 ~ 6.08)**	4.35 (3.16 ~ 5.98)**	2.44 (1.72 ~ 3.45)**
A large number	3.91 (2.98 ~ 5.13)**	3.90 (2.95 ~ 5.16)**	2.39 (1.75 ~ 3.25)**	9.03 (6.12 ~ 13.34)**	9.09 (6.11 ~ 13.54)**	3.56 (2.24 ~ 5.64)**

OR_u_: Univariate odds ratios

OR_a_: Adjusted odds ratios; adjusted for all significant variables listed in Table 2 plus the school variable

OR_m_: Multivariate odds ratios obtained by entering all the variables into the same logistic regression models, controlling for the significant background variables listed in Table 2 plus the school variable

*p value < 0.05; **p value < 0.01

N.A. The variables were not used to fit the multiple logistic regression model

As specified by the HBM, the adjusted analysis (adjusted for background factors that were significantly associated with IA) showed that higher perceived barriers for reducing Internet use (males: ORa = 1.26, 95 % CI = 1.23 to 1.29; females: ORa = 1.36, 95 % CI = 1.32 to 1.40) and higher perceived social benefits of Internet use (males: ORa = 1.19, 95 % CI = 1.17 to 1.22; females: ORa = 1.23, 95 % CI = 1.20 to 1.26) were positively associated with IA, while higher perceived self-efficacy for reducing Internet use (males: ORa = 0.66, 95 % CI = 0.64 to 0.69; females: ORa = 0.56, 95 % CI = 0.53 to 0.59) was negatively associated with IA.

Some scales showed significant associations in the adjusted analysis but the direction of the association was different from that specified by the HBM. According to the HBM, high perceived susceptibility and perceived severity related to IA would result in perceived threat, which would defer people from Internet overuse and avoid the development of IA. The two variables were hence expected to be negatively associated with IA. In contrast, we found that perceived susceptibility (significant among both males [ORa = 2.27, 95 % CI = 2.10 to 2.46] and females [ORa = 3.41, 95 % CI = 3.05 to 3.82]) and perceived severity of IA (significant among males [ORa = 1.14, 95 % CI = 1.07 to 1.21] but not females [ORa = 1.01, 95 % CI = 0.94-1.10]) were positively associated with IA. Similarly, it was expected that perceived cue to action to reduce Internet use would reduce Internet use and would hence be negatively associated with IA. We however, found a significant positive association between perceived cue to action and IA (males: ORa = 1.36, 95 % CI = 1.30 to 1.41; females: ORa = 1.47, 95 % CI = 1.40 to 1.55). Such unexpected associations possibly reflect causality issues due to the cross-sectional nature of the study, as those who were classified as IA would be more likely to perceive high susceptibility and high severity with respect to IA, and would be more likely to have been reminded by their parents, teachers, and/or social workers that they should reduce Internet use.

Adjusted for the significant socio-demographic factors, PNPIA was also significantly associated with IA for both males (*quite a number*: ORa = 2.85, 95 % CI = 2.21 to 3.67; *a large number*: ORa = 3.90, 95 % CI = 2.95 to 5.16) and females (*only a few*: ORa = 1.50, 95 % CI = 1.10 to 2.06; *quite a number*: ORa = 4.35, 95 % CI = 3.16 to 5.98; *a large number*: ORa = 9.09, 95 % CI = 6.11 to 13.54), when the ‘*nil*’ category was used as the reference group (Table 4).

### Correlations between HBM constructs and PNPIA

PNPIA was significantly correlated with the three constructs (perceived barriers for reducing Internet use, perceived social benefits of Internet use, and perceived self-efficacy for reducing Internet use) that were found to be statistically significant with IA for both males and females in the adjusted analysis and concurred with the specification of the HBM (Spearman correlation coefficients were respectively 0.17, 0.17, and −0.14, *p* < 0.001, among males and 0.22, 0.22, and −0.17, *p* < 0.001, among females [data not tabulated]).

### Variables of the HBM as potential mediators of the association between PNPIA and IA

The three variables of the HBM (perceived social benefits, perceived barriers and perceived self-efficacy), which were found to be significantly associated with IA in the adjusted analysis, and PNPIA were entered in the same multiple logistic regression model (Table 4). The results showed that PNPIA remained statistically significant among both females (*quite a number*: ORm = 2.44, 95 % CI = 1.72 to 3.45; *a large number*: ORm = 3.56, 95 % CI = 2.24 to 5.64) and males (*quite a number*: ORm = 2.07, 95 % CI = 1.57 to 2.73; *a large number*: ORm = 2.39, 95 % CI = 1.75 to 3.25). However, its adjusted odds ratios (ORa) diminished substantially as compared to those of the multiple logistic regression models containing only PNPIA (Table 4). For instance, the odds ratios decreased from 3.90 to 2.39 for males and 9.09 to 3.56 for females when comparing the categories of ‘*a large number*’ versus ‘*nil*’ for the variable on PNPIA. The three variables that followed HBM specifications therefore partially mediated the association between PNPIA and IA.

## Discussion

About one sixth of the sampled students were classified as IA cases. The prevalence was higher than that in mainland China [10, 13] (2.4 to 8.8 %, according to Young’s criteria) but was slightly lower than that in Taiwan (20.7 %, according to CIAS) [12]. Another local study, based on the 10-item Young’s Chinese Internet Addiction scale, reported prevalence of IA of 19.1 % among primary and secondary schools in 2008 [16]. The prevalence of mobile phones with Internet applications in the Hong Kong general population aged 15 and over was 62 % in 2012 [66] and about 80 % in 2014 (unpublished data). The prevalence of ownership of such phones was 74 % among our participants. Such high prevalence may speed up the spread of IA among adolescents; a hypothesis to be tested in future studies.

Our study found a statistically significant but mild sex difference in IA prevalence, with more male students classified as IA than female students. Shek’s study conducted in Hong Kong reported that male and female students had similar risk of having IA [18]. Similarly, the sex difference was also about 3 % (4.04 and 0.84 % for males and females) in China [10] and about 2.5 to 4.2 % in some European studies (6.2 to 6.7 % for males and 2 to 4.2 % for females) [7, 9]. Hence, the sex difference in IA prevalence detected in this and other studies was smaller than that of the prevalence of other risk behaviors among Asian students, such as smoking (28 and 3 % in Beijing [67], and 16.9 and 7.0 % in Korea [27]) and alcohol use (33.6 and 13.1 % in Guangdong [68]). Risk behaviors often involve cultural connotations such as masculinity, braveness and adventure [69, 70], but health professionals and parents should keep in mind that IA is not only confined to male secondary students. We hypothesized that Internet use would be less gender biased as compared to smoking and alcohol use, as Internet use is related to new technologies, daily functions, and applications. It is warranted to test this hypothesis in future studies.

It is seen that IA increases with school grade, suggesting the presence of an age effect. However, we did not measure age in this study. Importantly, the prevalence of IA among Secondary 1 (7^th^ year of formal education) students was already close to 10 %. Therefore, the age of onset of IA may be younger than that of other unhealthy behaviors, such as smoking [70]. IA is associated with multi-dimensional problems such as depression and academic behaviors [5, 20, 71]. Its negative impact on young adolescents requires special attention as lasting harms could result. Early detection and early intervention for IA among students are warranted.

We found that single parenthood was a risk factor of IA, especially for those living only with the father. In Chinese culture, maternal influences are stronger than that of paternal influences [72]. Previous studies reported that adolescents of single-parent families showed higher risks of substance abuse, drinking and smoking, especially among those living with only the father [73]. The differential impact of living with the father versus the mother may be mediated through the trend that mothers are more likely than fathers to exercise control on their children [74]. Interventions on IA hence need to pay special attention to single-parent families, especially to those involving a single father. Maternal education level, as compared to paternal education level, also showed stronger association with IA, again suggesting the presence of stronger maternal influence over paternal influence on IA. Since interventions for prevention of IA may require family participation [11, 75], the relative strength of maternal versus paternal influences is potentially important.

To our knowledge, this is the first study applying the HBM to explain IA. In this study, perceived susceptibility to IA in the next year among non-IA cases was noticeable. However, we found that perceived severity of consequences of IA was relatively low as half of the IA cases did not perceive that IA would be harmful to himself/herself. Furthermore, perceived susceptibility and perceived severity were found to be positively associated with IA, despite negative associations expected by the HBM. The discrepancy may be due to causality issues in the cross-sectional study design, which have been observed in other studies involving the HBM e.g., [76]. That is, those classified as having IA might be likely to recognize or experience some symptoms related to IA and hence in the absence of diagnosis, perceive that they would develop IA in the future, without knowing that they would have been classified as IA according to an external instrument. Furthermore, although health care workers worry about IA among adolescents, adolescents themselves might not see IA as problematic, as seen from our data. That may also explain why perceived severity was not negatively associated with IA, as some of those with IA might even see heavy use of the Internet as beneficial rather than harmful. Although some theories, such as those related to fear appeal [77], have indicated that perceived susceptibility and severity would result in perceived threat which may deter adoption of a risk behavior [77], our results suggest that IA prevention programs emphasizing the threat (perceived susceptibility and severity) associated with IA are unlikely to be effective. Therefore, fear appeal approaches [78], which have commonly been used for prevention of other risk behaviors (e.g., substance use), may not be applicable for prevention of IA among students.

The adolescents’ reliance of the Internet may have become a barrier against reducing its use, as over half of them agreed with at least one type of perceived barrier for reducing Internet use (i.e., reduced communication with friends, feeling lost, bored and old fashion). Internet use may have become an “indispensable” means of social interaction and even an internalized identity of the student [79]. In addition, perceived barriers were significantly and strongly associated with IA. Future intervention programs should hence consider how to remove such barriers. For example, school-based campaigns which encourage controlled Internet use and organize regular offline social activities for students may lower their perceived barriers for reducing Internet use and thus risk for developing IA [80].

Consistently, strong social benefits of Internet use were perceived by the sampled students, as about 60 % of them reported at least one type of perceived social benefit. They may see Internet use as a key means of developing and maintaining friendships and obtaining instrumental and emotional support. The Internet has hence become an important resource, supporting the formation of new social networks and establishing social capital among students [4]. According to social marketing principles, a behavior will be initiated if the perceived benefit exceeds the perceived cost [81], which is likely to be true for the case of Internet use among students. Health workers hence cannot underestimate difficulties for preventing or treating IA problems among students.

Given that Internet use may provide strong perceived and/or actual benefits, students should be trained to control their Internet use. As expected, low perceived self-efficacy to reduce Internet use was significantly associated with IA. It is hence important to increase students’ perceived self-efficacy to regulate their own Internet use [20, 25, 82]. School-based training like impulse-control techniques, time management and interpersonal communication skills have shown to be effective in improving perceived control over time and preventing Internet overuse [83].

Contrary to our hypothesis based on the HBM, the Cue to Action Scale (i.e., reminders from parents and teachers/social workers) to reduce Internet use was positively associated with risk of IA. It is likely that those with IA were often reminded by their parents, teachers and social workers to reduce Internet use. Hence, IA might be a cause of parental reminders instead of the reverse that parental reminders are a cause of IA. Again, similar reversed causality problems have been reported when applying the HBM to other cross-sectional studies [36]. However, it is important to point out that about one third of the IA cases reported that they had not been reminded by their parents to reduce Internet use. Previous studies have shown that parental control is protective against IA [84]. Parents should hence be trained on how to recognize their child’s IA symptoms, communicate with their child on IA issues, and exercise appropriate control on his/her Internet use.

We further observe that the presence of peers with IA was very common. Males, as compared to female students, perceived more peers with IA. The sex difference is consistent with the observation that males have higher IA prevalence than females. However, the relatively small sex difference in IA (<3 %) could at most provide a partial explanation of the higher PNPIA among males. It is also possible that male students with very high intensity of Internet use might be more likely than females to be clustered together to play online games or perform other online activities, or be more aware of one’s peers’ Internet use. Such speculations need to be confirmed by future studies. We did not define IA when asking the question about PNPIA as the definition is a complicated one and the question was asked prior to asking the items of CIAS. It is true that the participants may have different definitions about IA, but as the term is used colloquially in Hong Kong, it is likely that our participants were referring to peers that were likely to have apparent problematic Internet use. Therefore, the variable of PNPIA may be assessing such a perception, which is a potential source of peer influence on students’ IA status. Peer influence is one of the strongest predictors of risk behaviors among adolescents [41–45].

We also found that PNPIA was strongly associated with IA. Similar findings have not been reported in literature. Compared to adults, adolescents are less psychosocially mature [85] and more prone to peer influences during the cognitive processes of decision making regarding their risk behaviors [86]. Clustering and inducement effects may co-exist in this case [54]. Students’ beliefs about the outcomes of reducing one’s Internet use may be directly adopted from other peers with IA, or students with IA may meet and become friends with other IA peers via the Internet. This study further contributes to the understanding of the indirect effect of PNPIA on IA development by identifying three partial mediators that were related to the HBM. As mentioned, we cannot assume adolescents with or without IA would perceive IA as harmful; they might even see it as beneficial. Unlike the case of having peers who are drug users or smokers, some adolescents and even their family members might not perceive any need to avoid peers with IA. Thus, there was no obvious obstacle filtering adolescents’ exposure to influences on Internet use exerted by peers with IA. Such influences might operate through mediators including increases in perceived social benefits of Internet use and perceived barriers for reducing Internet use, and decreases in perceived self-efficacy for reducing Internet use, resulting in an increased risk of developing IA. Interventions may consider modifying these constructs.

The students’ social context may offer some potential explanations for observed mediating effects. To the students, having peers with IA may increase perceived social benefits of Internet use as they can connect with their peers with IA in the cyber world. These heightened perceived social benefits could increase Internet use and may lead to IA. Similarly, having peers with IA may increase the perceived barriers for reducing IA, as reduction of such use may result in potential loss of approval from and connection with those peers with IA, and hence “the world”. The contention that students connect with some peers with IA via the Internet therefore explains the mediating effects of the two constructs related to perceived social benefits of Internet use and perceived barriers for reducing Internet use. Furthermore, the presence of IA peers may adversely influence students’ subjective judgment on self-competence in controlling Internet use through vicarious learning [57].

The mediating effects have important implications for designing interventions. Ability to change perceptions on the three partial mediators of the associations between PNPIA and IA may neutralize potential influences of peers with IA on IA development among secondary school students. The findings of this study suggest the usefulness of group cognitive-behavioral interventions [83], which can involve both IA cases and their peers with IA. Such interventions may focus on altering their cognitions of the three specific constructs of the HBM. Given the strong effect of peer influence, changing relevant cognitions related to IA through group interventions (e.g., class discussion) may be a good entry point for preventing IA among students. Peer education programs led by those peers who have recovered from IA and act as role models are also potentially useful alternatives for changing perceived benefits and barriers related to Internet use, as well as promoting perceived self-efficacy toward reducing Internet use. However, caution should be exercised when implementing group interventions involving unidentified IA cases, as such events may connect such non-IA cases with new and old peers with IA, and expose non-IA cases to negative cognitive influences from such peers with IA, hence increasing their risk of developing IA.

It is important to point out that the partial but not full mediation effect implies that other mechanisms may exist in explaining the association between students’ PNPIA and IA. We have mentioned potential clustering effects of students with IA as one of the potential mechanisms. Modeling effects and emotional factors may also be involved [87, 88]. This is the first study attempting to discern such mediation effects. Future studies are warranted and allow us to understand better the mechanisms of peer influences on risk behaviors among students.

The study had some limitations. First, identification of IA and PNPIA relied solely on self-reported data and reporting bias may exist. In particular, the way that PNPIA was measured by one single question presents a limitation, as participants may have different understandings on the definition of IA and they might have referred to their responses as heavy Internet use or overuse, instead of IA. We were hence actually assessing perception instead of the true number of peers with IA. However, as IA is commonly used in daily language among adolescents in Hong Kong, the participants seemed to show no problem answering the question. We believe that PNPIA was a reasonable indicator of potential peer influences on Internet use that might increase students’ Internet use and hence risk of developing IA. Second, it was a cross-sectional survey, and no causal relationship could be established, as seen from the findings that some significant associations did not concur with the HBM. Third, a panel was formed to construct some scales to assess cognitions related to Internet use based on the HBM since such scales were not available. We restricted ourselves to social benefits when assessing perceived benefits of Internet use because social media is growingly influential, but other aspects of perceived benefits could have been used. Some constructs were only measured by one or two items (e.g., perceived susceptibility and perceived severity) and reliability of those indicators could not be assessed. One of the limitations is that the subscales formed had an uneven number of items, according to the panel’s suggestions. Last, we did not ask about age as we thought that age was relatively homogeneous with school grades. However, age might interact with the associations found due to maturation of the students. Future studies should investigate whether associations found in this study vary as age increases.

## Conclusions

With a large and representative sample, we found high prevalence of IA among Chinese secondary school students in Hong Kong. The results supported that specific constructs of the HBM (perceived social benefits of Internet use, perceived barriers for reducing Internet use and perceived self-efficacy to reduce Internet use) were risk factors of IA. We found that PNPIA was another significant factor, and more importantly, its influence on IA was partially mediated by the three aforementioned constructs of the HBM. We anticipated and discussed about huge difficulties and challenges expected when designing IA prevention programs, as students’ perceived benefits of Internet use might well exceed that of perceived cost. The cost-benefit imbalance also explains the relatively high IA prevalence observed in this sample. Interventions need to consider family input, cognitive changes and peer influences. Efforts for prevention of IA are uphill, as Internet use has penetrated into the essential aspects of adolescents’ daily life, including but not limited to learning, entertainment, social interactions and information seeking. Unfortunately, it seems that the IA problem may escalate further.
